# Association of Impaired Fasting Blood Glucose With Triple Coronary Artery Stenosis and Myocardial Infarction Among Patients With Coronary Artery Stenosis

**DOI:** 10.3389/fcell.2022.820124

**Published:** 2022-03-03

**Authors:** Chang Chen, Yequn Chen, Jiaxin Xiao, Yanhong Zhang, Zhaorui Yang, Peixuan Yang, Nan Lu, Kaihong Yi, Xiaojun Chen, Shaoxin Chen, Mary Clare O'Gara MSc, Michael O'Meara, Shu Ye, Xuerui Tan

**Affiliations:** ^1^ The First Affiliated Hospital of Shantou University Medical College, Shantou, China; ^2^ Shantou University Medical College, Shantou, China; ^3^ Department of Cardiovascular Sciences and NIHR Leicester Biomedical Research Centre, University of Leicester, Leicester, United Kingdom

**Keywords:** impaired fasting blood glucose, coronary artery disease, myocardial infarction, triple-vessel disease, single- or double-vessel disease

## Abstract

**Background:** The association between impaired fasting glucose level (IFG) and coronary heart disease (CAD) remain controversial. In the present study, we sought to ascertain a relationship of IFG with the number of diseased coronary artery and occurrence of myocardial infarction, among CAD cases.

**Methods:** We studied 1,451 consecutive no-diabetic patients who underwent coronary angiography at the First Affiliated Hospital of Shantou University Medical College in Southern China. Demographic, biochemical, clinical and angiographic data were collected.

**Results:** The prevalence of IFG was higher in patients with angiographically confirmed CAD than in subjects without angiographic evidence of CAD (33.4 *versus* 28.2%, *p* = 0.034). Compared with CAD cases without IFG, CAD cases with IFG had a higher odds ratio (OR) of having triple-vessel disease as opposed to having single- or double-vessel disease [OR = 1.53, 95% confidence interval (CI) = 1.13–2.07]. Furthermore, the occurrence of MI was higher in CAD cases with IFG than in CAD cases without IFG (OR = 1.73, 95% CI = 1.27–2.36).

**Conclusions:** There is an association between IFG and a predisposition to severe CAD indicated by triple vessel disease or myocardial infarction.

## Introduction

Many epidemiological studies have shown that individuals of diabetes mellitus are more likely to have coronary artery disease (CAD) than no-diabetic individuals (Kannel and McGee, 1979; Martins et al., 2015). It is now widely accepted that diabetes mellitus is a major risk factor for CAD.

Impaired fasting glucose (also known as impaired fasting glycemia, IFG) is a type of prediabetes. In individuals with IFG, fasting blood sugar levels are consistently above the normal range but below the threshold for a diagnosis of diabetes mellitus. IFG has been associated with micro vascular disease (Hanna-Moussa et al., 2009), heart failure and cardiovascular mortality (Henry et al., 2002; Sorkin et al., 2005; Xu et al., 2015; Huang et al., 2016; Cai et al., 2020; Cai et al., 2021; Mai et al., 2021) Several meta-analyses concluded that IFG was associated with an increased risk of CAD. However, it is still unclear as to whether or not there is a relationship between IFG and severity of CAD (Yan et al., 2009; Konstantinou et al., 2010), and further investigations are required.

In the present study, we examined a cohort of non-diabetic individuals undergoing diagnostic or interventional coronary angiography and ascertained whether there was an association of IFG with angiographically confirmed CAD, with the extent of atherosclerosis in CAD patients, and/or with the occurrence of myocardial infarction (MI) among CAD cases.

## Subjects and Methods

### Subjects

The patients scheduled to undergo clinically indicated diagnostic or therapeutic coronary catheterization at the First Affiliated Hospital of Shantou University Medical College during the period from 1 January 2009 to 31 December 2013 were recruited in this study. Patients who were currently pregnant or under taking fasting plasma glucose-lowering or steroid hormones medication or with history of chronic kidney disease and diabetes were excluded. A total of 1,451 non-diabetic individuals were enrolled in this study. All subjects were Chinese and older than 18 years. The study was approved by the research ethics committee of the First Affiliated Hospital of Shantou University Medical College.

Demographic and clinical data including age, sex, total cholesterol (TC) level, low-density-lipoprotein-cholesterol (LDLc) level, high-density-lipoprotein-cholesterol (HDLc) level, triglycerides level, blood pressure, coronary angiographic findings, and prevalent or incident MI were collected from hospital records. Blood samples for TC, LDLc, HDLc, triglycerides and fasting blood glucose (FBG) measurements were collected after overnight fasting. TC, LDLc, HDLc, triglycerides and FBG measurements were conducted by the clinical chemistry department of the First Affiliated Hospital of Shantou University Medical College. Coronary angiography was carried out by experienced interventional cardiologists. CAD was defined as ≥50% diameter stenosis in any of the major epicardial coronary artery, as measured by quantitative coronary angiography (Douglas et al., 2011; Dehmer et al., 2012; Fihn et al., 20142014). Disease severity was determined by number of diseased vessels (Golbahar et al., 2005). In this study, myocardial infarction included acute myocardial infarction and history of myocardial infarction. A diagnosis of acute myocardial infarction was established when there is acute myocardial injury with clinical evidence of acute myocardial ischemia and with detection of a rise and/or fall of cTn values with at least one value above the 99th percentile URL and at least one of the following: symptoms of myocardial ischemia; new ischemic ECG changes; development of pathological Q waves; imaging evidence of new loss of viable myocardium or new regional wall motion abnormality in a pattern consistent with an ischemic etiology; identification of a coronary thrombus by angiography. (Thygesen et al., 2018) IFG was defined by a fasting glucose level of 5.61–6.99 mmol/L and non-IFG by ≤ 5.60 mmol/L.

### Statistical Analysis

Data were analyzed using SPSS software, version 21.0 (SPSS Inc., Chicago, IL). The T-test and analysis of variance (ANOVA) were used to determine any differences in age, systolic blood pressure, diastolic blood pressure, TC level, LDLc level, HDLc level, and triglycerides level, between study groups. The χ2 test was performed to compare male/female ratio between groups and to compare the presence/absence of IFG between groups. Logistic regression analyses were performed to ascertain a relationship between IFG and the number of coronary artery with ≥50% stenosis among CAD cases with or without adjustment for age, TC and LDLc, and to test a relationship between IFG and MI among CAD cases with or without adjustment for sex, systolic blood pressure, diastolic blood pressure and triglyceride. All p-values are two sided.

## Results

Demographic and clinical characteristics of the study subjects are shown in Table 1

**TABLE 1:** Demographic and clinical characteristics of study subjects.

	Number of coronary artery with ≥50% stenosis					Occurrence of MI in CAD cases^a^		
	0 (*n* = 586)	1 or 2 (*n* = 601)	3 (*n* = 264)	*p*-value^b^	*p*-value^c^	No (*n* = 296)	Yes (*n* = 569)	*p*-value
Sex (male/female)	321/265 (54.8%/45.2%)	484/117 (80.5%/19.5%)	216/48 (81.8%/18.2)	⟨0.001	0.658	218/78 (73.6%/26.4%)	482/87 (84.7%/15.3%)	⟨0.001
Age (years)	58.25 ± 10.86	62.10 ± 11.18	65.59 ± 10.67	⟨0.001	⟨0.001	63.64 ± 10.54	62.92 ± 11.44	0.358
Systolic blood pressure (mmHg)	135.54 ± 21.38	134.15 ± 24.61	133.44 ± 23.00	0.193	0.679	141.02 ± 22.34	130.24 ± 24.20	⟨0.001
Diastolic blood pressure (mmHg)	83.17 ± 13.56	82.07 ± 14.21	81.31 ± 13.30	0.070	0.447	84.07 ± 13.29	80.68 ± 14.13	0.001
Total cholesterol (mmol/L)	4.91 ± 1.18	4.86 ± 1.24	5.19 ± 1.45	0.445	0.001	5.01 ± 1.45	4.94 ± 1.24	0.474
Low density lipoprotein (mmol/L)	3.07 ± 0.96	3.06 ± 1.04	3.33 ± 1.15	0.176	0.002	3.14 ± 1.17	3.15 ± 1.04	0.830
High density lipoprotein (mmol/L)	1.17 ± 0.33	1.13 ± 0.30	1.12 ± 0.27	0.006	0.727	1.15 ± 0.30	1.12 ± 0.29	0.137
Triglyceride (mmol/L)	1.56 ± 1.03	1.60 ± 1.05	1.51 ± 0.88	0.227	0.510	1.72 ± 1.25	1.49 ± 0.83	0.019
Impaired fasting glucose^d^ (Yes/No)	165/421 (28.2%/71.8%)	183/418 (30.4%/69.6%)	106/158 (40.2%/59.8%)	0.034	0.005	76/220 (25.7%/74.3%)	213/356 (37.4%/62.6%)	0.001

Continuous variables are presented as mean ± standard deviation, and categorical variables are expressed as frequency and percentage.

a Referred to cases with ≥50% stenosis in at least 1 coronary artery.

d Fasting blood glucose level = 5.61–6.99 mmol/L.

b Comparing subject without ≥50% stenosis to subjects with ≥50% stenosis in at least 1 coronary artery.

c Comparing subject with ≥50% stenosis in 1 or 2 coronary arteries to subjects with ≥50% stenosis in 3 coronary arteries.

The prevalence of IFG (fasting blood glucose level 5.61–6.99 mmol/L) was higher in patients with angiographically confirmed CAD (≥50% stenosis in at least 1 coronary artery) than in individuals without angiographic evidence of CAD (33.4 versus 28.2%, *p* = 0.034 without adjustment for co-variants, *p* = 0.044 with adjustment for sex, age and HDLc level).

Compared with CAD cases without IFG, CAD cases with IFG had a higher odds ratio (OR) of having triple-vessel disease (≥50% stenosis in 3 coronary arteries) as opposed to having single- or double-vessel disease (≥50% stenosis in just 1 or 2 coronary arteries) [OR = 1.53, 95% confidence interval (CI) = 1.13-2.07], which remained significant after adjusting age, total cholesterol level, and LDLc level (OR = 1.52, 95% CI = 1.10-2.10) (Table 2).

**TABLE 2:** Impaired fasting blood glucose and extent of coronary artery disease.

Impaired fasting glucose	Numbers of coronary artery with ≥50% stenosis	Odds ratio (95% CI)	Adjusted odds ratio (95% CI)^a^	
	1 or 2	3		
No^b^	418 (69.6%)	158 (59.8%)	1.00 (reference)	1.00 (reference)
Yes^c^	183 (30.4%)	106 (40.2%)	1.53 (1.13–2.07)	1.52 (1.10–2.10)

a Logistic regression analysis with adjustment for age, total cholesterol level and Low density lipoprotein.

b Fasting blood glucose level ≤5.60 mmol/L.

c Fasting blood glucose level 5.61–6.99 mmol/L.

Furthermore, the occurrence of MI was higher in CAD cases with IFG than in CAD cases without IFG (OR = 1.73, 95% CI = 1.27-2.36), which remained significant after adjustment for sex, age, systolic blood pressure, diastolic blood pressure and triglyceride) (OR = 1.79, 95%CI = 1.28-2.50) (Table 3).

**TABLE 3:** Impaired fasting glucose and myocardial infarction In patients with ≥50% stenosis in at least one coronary artery.

Impaired fasting glucose	Myocardial infarction		Odds ratio (95% CI)	Adjusted odds ratio (95%CI)^a^
	No	Yes		
No^b^	220 (38.2%)	356 (61.8%)	1.00 (reference)	1.00 (Reference)
Yes^c^	76 (26.3%)	213 (73.7%)	1.73 (1.27–2.36)	1.79 (1.28–2.50)

a Logistic regression analysis with adjustment for sex, systolic blood pressure, diastolic blood pressure and triglyceride level.

b Fasting blood glucose level ≤5.60 mmol/L.

c Fasting blood glucose level 5.61–6.99 mmol.

## Discussion

In our study, we found that the prevalence of IFG was higher in patients with angiographically confirmed CAD than in subjects without angiographic evidence of CAD. It was in agreement with previous study reporting and several meta-analysis (Konstantinou et al., 2010; Xu et al., 2015; Huang et al., 2016; Cai et al., 2020).

Previous studies had shown that prediabetes were associated with an increased risk of heart failure (HF), and a worse prognosis with HF. (Cai et al., 2021; Mai et al., 2021) Also, In our study, we found that compared with CAD cases without IFG, CAD cases with IFG were suffered from triple-vessel stenosis more than single- or double-vessel stenosis. It may be suggested that IFG promoted involvement of coronary arteries with a more aggressive and diffused atherosclerotic process and had a worse prognosis.

It is still unclear how IFG promoted the progress of CAD. Hyperglycemia, vascular insulin resistance and other inflammatory cytokines contributed to endothelial vasodilator dysfunction and increased formation of Extracellular Matrix in IFG patients (Wasserman et al., 2018). Advanced glycation products (AGEs) stimulated monocytes to secrete inflammatory cytokines and result in the formation of foam cells (Vlassara et al., 1988; Jinnouchi et al., 1998; Wasserman et al., 2018). All of these factors may contribute to progression of the triple-vessel disease in IFG patients.

Several limitations should be considered. Firstly, the cross-sectional nature of the study prevents us from confirming the causal effects angiographically confirmed CAD cases in the pathogenesis of IFG. Secondly, triple-vessel disease often indicates a poor prognosis for patients. However, in this study, we did not observe the prognosis of the patients which limited its persuasion. Third, not only IFG but also impaired glucose tolerance (IGT), plays important role in CAD progression (Huang et al., 2016). However, in the study, we did not include IGT which need to be further investigated.

## Data Availability

The raw data supporting the conclusions of this article will be made available by the authors, without undue reservation.
